# Enhanced killing of SCC17B human head and neck squamous cell carcinoma cells after photodynamic therapy plus fenretinide via the *de novo* sphingolipid biosynthesis pathway and apoptosis

**DOI:** 10.3892/ijo.2015.2909

**Published:** 2015-02-26

**Authors:** NITHIN B. BOPPANA, URSULA STOCHAJ, MOHAMED KODIHA, ALICJA BIELAWSKA, JACEK BIELAWSKI, JASON S. PIERCE, MLADEN KORBELIK, DUSKA SEPAROVIC

**Affiliations:** 1 Department of Pharmaceutical Sciences, Eugene Applebaum College of Pharmacy and Health Sciences, Wayne State University, Detroit, MI 48201, USA; 2 Karmanos Cancer Institute, Wayne State University, Detroit, MI 48201, USA; 3 Department of Physiology, McGill University, Montreal, QC H3G 1YC, Canada; 4 Department of Biochemistry and Molecular Biology, Medical University of South Carolina, Charleston, SC 29425, USA; 5 British Columbia Cancer Agency, Vancouver, BC V5Z 1L3, Canada

**Keywords:** apoptosis, ceramide synthase, dihydroceramide, fenretinide, photodynamic therapy, sphingolipids

## Abstract

Because photodynamic therapy (PDT) alone is not always effective as an anticancer treatment, PDT is combined with other anticancer agents for improved efficacy. The clinically-relevant fenretinide [N-(4-hydroxyphenyl) retinamide; 4HPR], was combined with the silicon phthalocyanine photosensitizer Pc4-mediated PDT to test for their potential to enhance killing of SCC17B cells, a clinically-relevant model of human head and neck squamous cell carcinoma. Because each of these treatments induces apoptosis and regulates the *de novo* sphingolipid (SL) biosynthesis pathway, the role of ceramide synthase, the pathway-associated enzyme, in PDT+4HPR-induced apoptotic cell death was determined using the ceramide synthase inhibitor fumonisin B1 (FB). PDT+4HPR enhanced loss of clonogenicity. zVAD-fmk, a pan-caspase inhibitor, and FB, protected cells from death post-PDT+4HPR. In contrast, the anti-apoptotic protein Bcl2 inhibitor ABT199 enhanced cell killing after PDT+4HPR. Combining PDT with 4HPR led to FB-sensitive, enhanced Bax associated with mitochondria and cytochrome *c* redistribution. Mass spectrometry data showed that the accumulation of C16-dihydroceramide, a precursor of ceramide in the *de novo* SL biosynthesis pathway, was enhanced after PDT+4HPR. Using quantitative confocal microscopy, we found that PDT+4HPR enhanced dihydroceramide/ceramide accumulation in the ER, which was inhibited by FB. The results suggest that SCC17B cells are sensitized to PDT by 4HPR via the *de novo* SL biosynthesis pathway and apoptosis, and imply potential clinical relevance of the combination for cancer treatment.

## Introduction

Photodynamic therapy (PDT), a clinically-approved cancer therapy, utilizes a photosensitizer, a light at a wavelength corresponding to the photosensitizer’s absorbance, and molecular oxygen to produce reactive oxygen species in malignant cellular targets, and ultimately causing their destruction (1). Apoptosis is an important mechanism for eliminating tumors (2–6), and the effectiveness of PDT regimens correlates with tumor cell apoptosis (7). Because PDT itself is not always effective as a tumor treatment (8,9), PDT is combined with other anticancer agents for improved therapeutic benefit. 4HPR [N-(4-hydroxyphenyl) retinamide; fenretinide], a proapoptotic, clinically-relevant anticancer synthetic retinoid (10), is a potential candidate for combined treatment.

**Figure 1:**
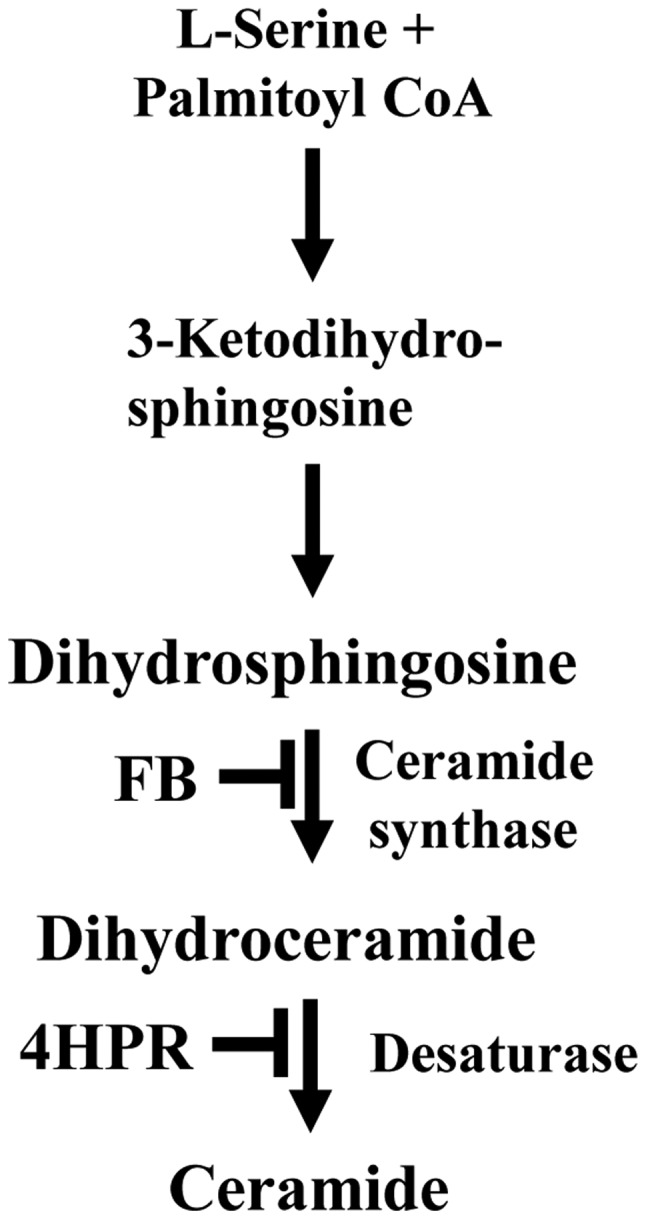
*De novo* SL biosynthesis pathway is FB- and 4HPR-sensitive.

As others and we have shown, sphingolipids (SLs), e.g., ceramide, generated via the *de novo* SL biosynthetic pathway (Fig. 1), have been implicated in apoptotic cell death after PDT and 4HPR in various malignant cell lines (11–14). Ceramide synthase catalyzes a reaction in the *de novo* SL biosynthesis pathway, in which a fatty acyl group is added to dihydrosphingosine to form dihydroceramide. Ceramide is formed in the subsequent desaturase-dependent reaction, which can be inhibited by 4HPR (15). The ceramide synthase inhibitor fumonisin B1 (FB) renders cells resistant to apoptosis after PDT and 4HPR (14,16).

There have been no reports on combining PDT with 4HPR for improving the efficacy of PDT. The objective of the present study was to test the hypothesis that combining PDT with 4HPR enhances cell killing via apoptosis and the *de novo* SL biosynthesis pathway. We used SCC17B cells, a human head and neck squamous cell carcinoma cell line, representing a model that is potentially PDT-treatable in the clinic (17).

## Materials and methods

### Materials

The phthalocyanine photosensitizer Pc4, HOSiPcOSi(CH_3_)_2_(CH_2_)_3_N(CH_3_)_2_, was kindly provided by Dr Malcolm E. Kenney (Department of Chemistry, Case Western Reserve University, Cleveland, OH, USA). 4HPR [N-(4-hydroxyphenyl) retinamide], fetal bovine serum and goat serum were purchased from Sigma-Aldrich (St. Louis, MO, USA). Cellgro DMEM/F-12 medium was obtained from Thermo Fisher Scientific (Waltham, MA, USA). Inhibitors were from the sources indicated in brackets: zVAD-fmk (MBL International Corp., Woburn, MA, USA), fumonisin B1 (Cayman Chemicals, Ann Arbor, MI, USA) and ABT-199 (Selleck Chemicals, Houston, TX, USA).

### Cell culture and treatments

SCC17B cells were obtained from Dr Thomas Carey (University of Michigan, Ann Arbor, MI, USA). Cells were grown in DMEM/F-12 medium containing 10% fetal bovine serum, 100 U/ml penicillin and 100 μg/ml streptomycin (Life Technologies, Carlsbad, CA, USA) in a humidified incubator at 37°C and 5% CO_2_. For all experiments, unless indicated otherwise, incubation of cells was carried out in a humidified incubator at 37°C and 5% CO_2_. All treatments, as well as staining with Mitotracker Red CMXRos (see below) were added to cells in growth medium. After overnight incubation with Pc4 (20 nM), 4HPR (2.5 μM) was added immediately prior to irradiation. Cells were irradiated at room temperature with red light (2 mW/cm^2^; λ_max_ ~670 nm) using a light-emitting diode array light source (EFOS, Mississauga, ON, Canada) at the fluence of 200 mJ/cm^2^ and incubated for 10 h. Phosphate-buffered saline (PBS) without calcium and magnesium was used for confocal microscopy. PBS containing calcium and magnesium was used for mass spectrometry (MS). Both types of PBS were purchased from Life Technologies.

### Clonogenic assay

Cell survival was assessed using clonogenic assay according to the modified pre-plating protocol, as we have described (18). Cells were resuspended in growth medium containing Pc4 (20 nM) and seeded (250 cells/well) in a 6-well plate (Thermo Fisher Scientific). After overnight incubation, the cells were irradiated. 4HPR was added immediately prior to irradiation. The inhibitors FB, zVAD-fmk (zVAD) and ABT-199 (ABT) were added 1 h prior to PDT±4HPR. After 14 days of incubation, the medium was aspirated, the plates were stained with crystal violet (0.1% in 20% ethanol; Sigma-Aldrich) for 30 sec, rinsed with water and air-dried. Colonies (≥50 cells) were counted using eCount Colony Counter (VWR International, Radnor, PA, USA). Plating efficiency was 36% (n=16).

### Quantitative confocal microscopy

Cells were grown on coverslips (Thermo Fisher Scientific) in 6-well plates (Thermo Fisher Scientific). To visualize mitochondria, treated cells were incubated with Mitotracker Red CMXRos (250 nM; Life Technologies) in growth medium for 30 min. After treatments, the coverslips were washed with cold PBS, and fixed by incubation for 15 min in 4% formaldehyde (Thermo Fisher Scientific) in PBS. After washing with PBS, cells were permeabilized with ice-cold acetone/methanol (1:1) for 10 min. After blocking with 3% goat serum and 3% fetal bovine serum in PBS for 1 h at 4°C, cells were incubated at room temperature for 45 min with mouse monoclonal anti-dihydroceramide/ceramide antibodies (1:30; ALX-804-196; Enzo Life Sciences, Ann Arbor, MI, USA). After washing with cold PBS, cells were incubated at room temperature for 45 min with Alexa 488-conjugated goat anti-mouse monoclonal IgM antibodies (1:200; 115-546-075; Jackson ImmunoResearch, West Grove, PA, USA) and washed again with cold PBS. To visualize the nuclei, cells were stained with 4′6-diamidino-2-phenylindole (DAPI; Life Technologies; 1 μg/ml in cold PBS) for 10 min at room temperature and washed with cold PBS. The coverslips were mounted on slides using the ProLong Antifade kit (P7481; Life Technologies). Zeiss LSM780 confocal microscope equipped with a 100×1.4 NA OIL DIC D objective (Carl Zeiss, Thornwood, NY, USA) was used for acquiring the images.

For ER and dihydroceramide/ceramide detection, mouse monoclonal anti-KDEL antibody (1:50; ab12223; Abcam, Cambridge, MA, USA) and anti-dihydroceramide/ceramide antibodies were combined with Alexa 594-conjugated goat anti-mouse IgG (115-585-071) and Alexa 488-conjugated goat anti-mouse IgM antibodies (both 1:200; monoclonal, from Jackson ImmunoResearch), respectively. For visualization of Bax, mouse monoclonal anti-Bax antibodies (1:50; ab5714; Abcam) were combined with Alexa 488-conjugated monoclonal goat anti-mouse IgG antibodies (1:200; 115-545-071; Jackson ImmunoResearch). For visualization of cytochrome *c* (cyt *c*) redistribution, mouse monoclonal anti-cyt *c* antibodies (1:50; 556432; BD Biosciences, San Jose, CA, USA) were combined with Alexa 594-conjugated goat anti-mouse IgG (1:200; Jackson ImmunoResearch). As a criterion to score the cells positive for the redistribution of cyt *c* the margin of cyt *c* staining around the nuclei greater than 10 μm was used. At least 100 cells were assessed for every condition in each experiment. Confocal microscopy imaging was performed at the Microscopy, Imaging and Cytometry Resources Core at Wayne State University, School of Medicine.

Quantifications were carried out as follows: dihydroceramide/ceramide fluorescence associated with the ER and the mitochondria, as well as Bax fluorescence associated with the mitochondria, were all quantified with MetaXpress software (version 5 5.00.20; Molecular Devices, LLC; Sunnyvale, CA, USA) (19). Mitotracker or anti-KDEL antibodies were employed to demarcate the mitochondria or the ER. Dihydroceramide/ceramide or Bax-pixel intensities associated with these subcellular compartments were measured with the multiwavelength cell scoring module. The correct compartment identification was verified by visual inspection. All images were corrected for background contribution before quantification. For Bax associated with mitochondria a minimum of 488 regions were measured for each data point. For dihydroceramide/ceramide associated with the ER and mitochondria a minimum of 680 or 518 regions were measured for each data point, respectively. Significant differences (p<0.05) were determined using comparison of multiple samples by one-way ANOVA.

### Electrospray ionization/double mass spectrometry (MS) analysis

After treatments, cells were collected on ice, washed twice with cold PBS, resuspended in a mixture of ethyl acetate/methanol (1:1, v/v; EMD Millipore, Billerica, MA, USA), dried under nitrogen in the N-EVAP analytical evaporator (Organomation; Berlin, MA, USA), and shipped overnight on dry ice to the Lipidomics Shared Resource Facility (Medical University of South Carolina, Charleston, SC, USA) for further processing. After extraction, SLs were separated by high performance liquid chromatography, introduced to the electrospray ionization source and then analyzed by double MS using TSQ 7000 triple quadrupole mass spectrometer (Thermo-Fisher Scientific) as described previously (20). The pmoles of SLs were normalized per mg protein. Protein content was determined by a modified Bradford assay per manufacturer’s instructions (Bio-Rad, Hercules, CA, USA).

### Statistical analysis

Significant differences (p<0.05) were determined using Student’s t-test or one-way ANOVA.

## Results

### Enhanced cell killing after PDT+4HPR is FB-, zVAD-fmk- and ABT-199-sensitive

Clonogenic assay was used to test whether combining PDT with 4HPR sensitizes SCC17B cells to PDT. As shown in Table I, when PDT and 4HPR were used at LD20 each, i.e., the dose reducing survival by 20%, 63% of PDT+4HPR-treated cells were unable to form colonies. To determine whether apoptosis and ceramide synthase are necessary for enhanced cell killing after PDT+4HPR, we used the pancaspase inhibitor zVAD-fmk (zVAD), the Bcl2 inhibitor ABT-199 (ABT), and the ceramide synthase inhibitor FB (21–23). We have previously shown that FB and zVAD rendered cells resistant to PDT (16). In contrast, ABT sensitized cells to PDT (16). Apoptosis is a major pathway involved in the anti-cancer action of 4HPR (24) and the *de novo* SL biosynthesis pathway plays a role in 4HPR-induced apoptosis (14,25). As shown in Table I, all inhibitors were non-toxic (LD<5). FB and zVAD rendered the cells resistant not only to PDT and 4HPR alone, but also to PDT+4HPR. In contrast, ABT sensitized SCC17B cells to PDT±4HPR.

### PDT+4HPR-enhanced Bax associated with mitochondria and cyt c redistribution is inhibited by FB

**Figure 2:**
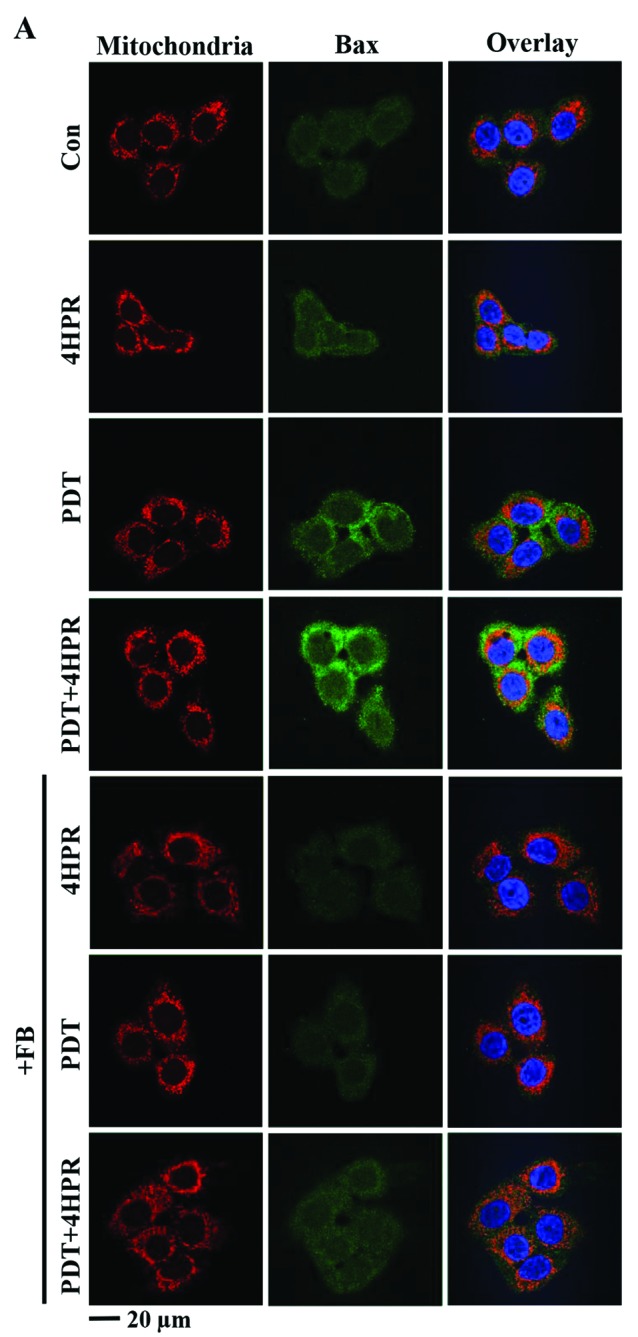

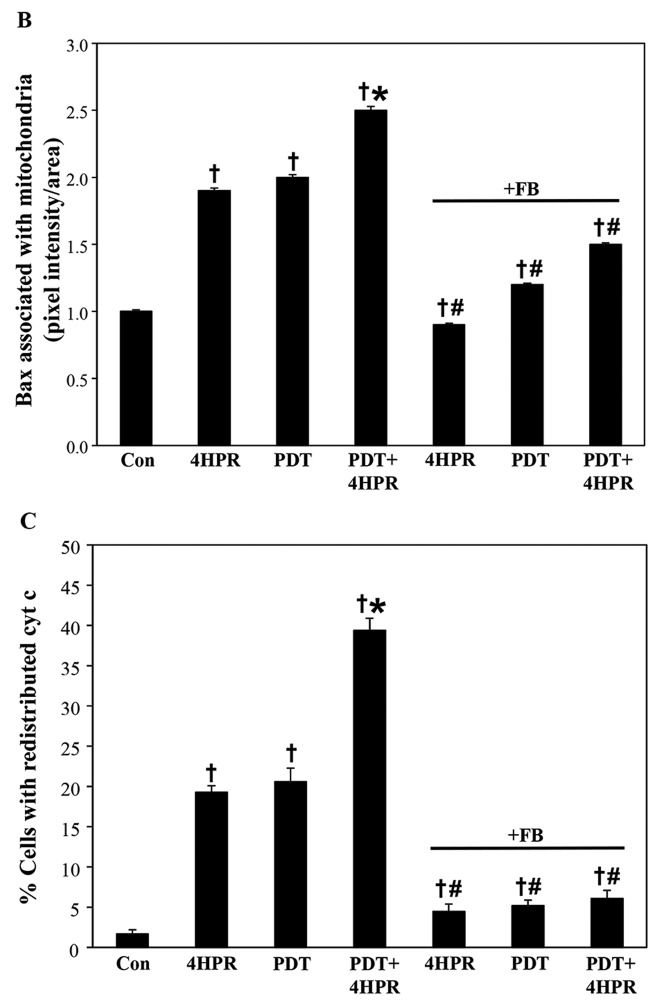
PDT+4HPR-induced enhanced Bax associated with mitochondria and cyt *c* redistribution are inhibited by FB. FB (10 μM) was added 1 h prior to PDT (20 nM Pc4 + 200 mJ/cm^2^; LD20), 4HPR (2.5 μM; LD20) or the combination. Incubation time was 10 h post-treatments. After treatments, cells were immunostained with anti-Bax antibodies (A and B) or anti-cyt *c* antibodies (C). Incubation with Mitotracker Red CMXRos was carried out prior to immunostaining with anti-Bax antibodies (A and B). Nuclei were visualized with DAPI. Images were acquired by confocal microscopy using identical settings. (B) Bax fluorescence located in the mitochondria was quantified with MetaXpress software. The graph shows Bax fluorescence/mitochondrial area. The data were normalized to the untreated control. Results are shown as the average ± SEM. (C) To calculate percentages of cells with redistributed cyt *c*, at least 100 cells were scored for every sample. Each bar indicates an average ± SEM from 3–4 samples. (B and C) Significant differences are shown between: ^†^treatment and untreated control; ^#^(treatment + inhibitor) and treatment; ^*^combination and individual treatments. Con, untreated control.

The mitochondrial apoptosis pathway, including Bax translocation to mitochondria and cyt *c* redistribution/release, is induced by PDT and 4HPR (16,26–28). Both of these processes are inhibited by FB after PDT (16). The question is whether the mitochondrial apoptosis pathway is affected by combining PDT with 4HPR, and whether the process is ceramide synthase-dependent. Using quantitative confocal microscopy we found that Bax associated with mitochondria and cyt *c* redistribution were induced after each individual treatment (Fig. 2). PDT+4HPR enhanced Bax associated with mitochondria and cyt *c* redistribution, and FB inhibited both processes.

### PDT+4HPR enhances C16-dihydroceramide, not ceramide, accumulation

**Figure 3:**
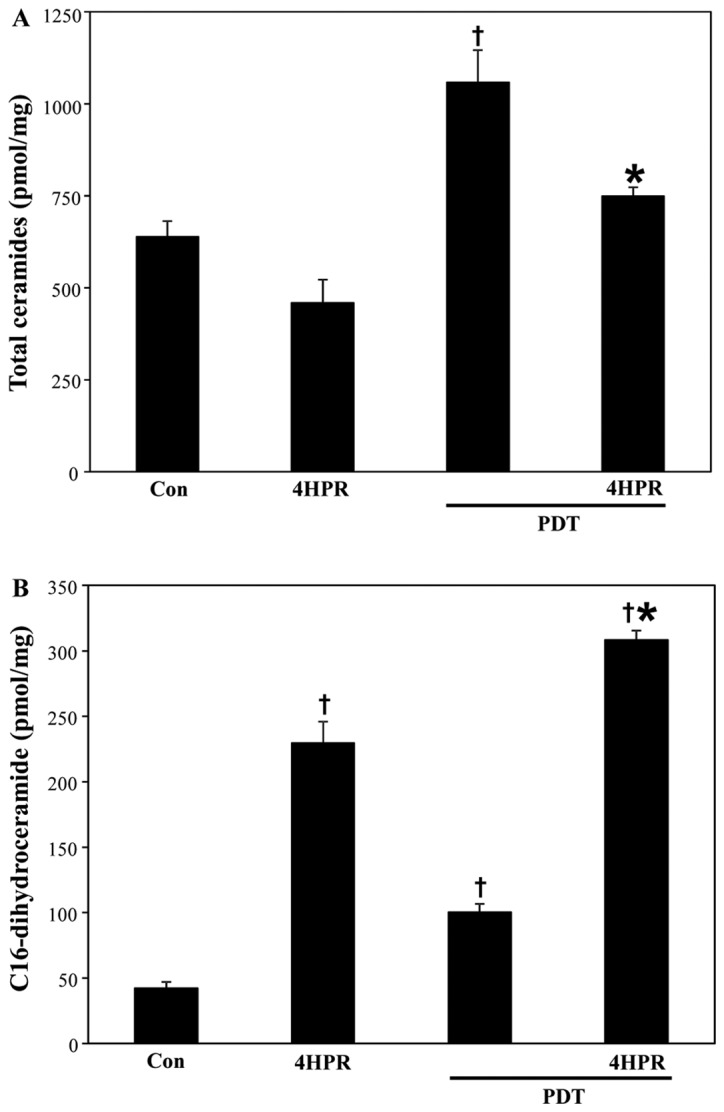
(A) Effect of PDT±4HPR on total ceramide levels. (B) PDT+4HPR enhances C16-dihydroceramide accumulation. Cells were treated with PDT (20 nM Pc4 + 200 mJ/cm^2^; LD20), 4HPR (2.5 μM; LD20) or the combination, incubated for 10 h, collected and processed for MS. The levels of SLs were calculated as pmoles/mg protein and are shown as the average ± SEM (n=3–4). Significant differences are shown between: ^†^treatment and untreated control; ^*^combination and individual treatments. Con, untreated control. PDT alone data from ref. 16.

Both PDT and 4HPR regulate the *de novo* SL biosynthesis pathway (12–16,29). The question is what the effects of combining PDT with 4HPR on the cellular SL profile are. We used MS to address the question. As depicted in Fig. 3A, in contrast to 4HPR, PDT increased total ceramide accumulation. Combining PDT with 4HPR attenuated PDT-induced increase in total ceramide levels. The accumulation of individual ceramides, by and large, followed the same pattern (Table II). 4HPR did not significantly raise the levels of any individual ceramide. In contrast, 4HPR increased accumulation of C16-dihydroceramide, a *de novo* SL biosynthesis pathway metabolite by 445% above basal levels (Fig. 3B). PDT also increased the levels of C16-dihydroceramide by 138% beyond resting levels. Combining PDT with 4HPR enhanced accumulation of C16-dihydroceramide by 632%.

### PDT+4HPR-induced enhanced ceramide accumulation in the ER is inhibited by FB

**Figure 4:**
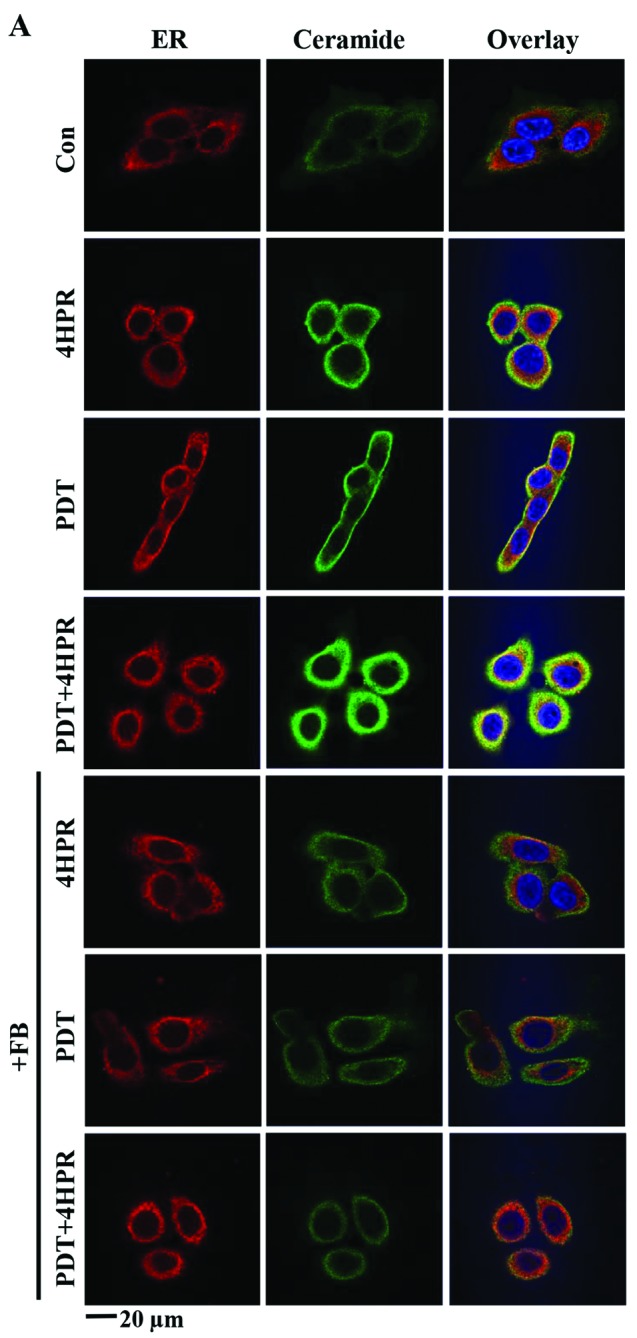

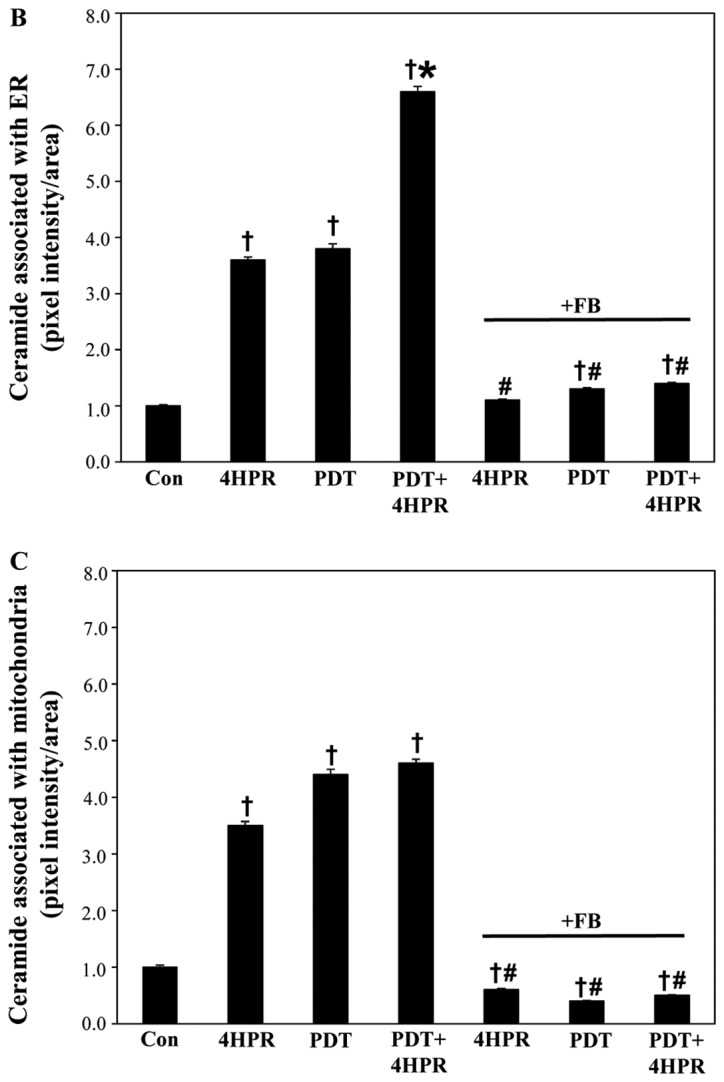
(A and B) PDT+4HPR-enhanced ceramide accumulation in the ER is inhibited by FB. (C) FB inhibits ceramide accumulation in mitochondria after PDT±4HPR. Cells were treated with FB (10 μM) 1 h prior to PDT (20 nM Pc4 + 200 mJ/cm^2^; LD20), 4HPR (2.5 μM; LD20), or the combination, incubated for 10 h, and immunostained with anti-ceramide and, with anti-KDEL antibodies (A, B). (C) Incubation with Mitotracker Red CMXRos was carried out prior to immunostaining with anti-ceramide antibodies. Nuclei were visualized with DAPI. All images were acquired by confocal microscopy with identical settings. MetaXpress software was used to quantify ceramide fluorescence located in the ER (B) and mitochondria (C). (B and C) Data are shown as the average ± SEM. The graphs depict ceramide fluorescence/ER area (B), or ceramide fluorescence/mitochondrial area (C). Results were normalized to the untreated control. Significant differences are shown between: ^†^treatment and untreated control; ^#^(treatment + inhibitor) and treatment; ^*^combination and individual treatments. Con, untreated control.

Because the *de novo* SL biosynthesis pathway is localized to the ER, we tested the effect of combining PDT with 4HPR on dihydroceramide/ceramide accumulation in the ER. Using an antibody that recognizes dihydroceramide and ceramide (30) and the ER marker anti-KDEL for quantitative confocal microscopy, we found that PDT+4HPR enhanced dihydroceramide/ceramide accumulation in the ER (Fig. 4A and B). FB inhibited ER-associated ceramide accumulation after PDT±4HPR.

We have shown that PDT-induced mitochondrial dihydroceramide/ceramide accumulation is FB-sensitive (16). However, it is unknown what impact on mitochondrial dihydroceramide/ceramide accumulation combining PDT with 4HPR might have. Using the same anti-dihydroceramide/ceramide antibody and the mitochondrial marker Mitotracker for quantitative confocal microscopy, we found that PDT and 4HPR alone did induce mitochondrial dihydroceramide/ceramide accumulation (Fig. 4C). However, the effect was not enhanced after PDT+4HPR. FB inhibited mitochondrial ceramide accumulation after all the treatments.

## Discussion

The present study is novel because, to our knowledge, no report exists on combining PDT with 4HPR. The results suggest that PDT+4HPR-induced enhanced killing of SCC17B cells depends on ceramide synthase, caspase activation and inhibition of Bcl2, and is associated with ceramide synthase-dependent mitochondrial apoptosis pathway. We have reported similar findings after PDT alone (16). In contrast, in breast cancer cells FB did not affect cell death after 4HPR (14). The discrepancy between the findings could be due to the use of different assays, i.e. clonogenic vs. short-term viability assay, respectively (31), or cell type-specificity of FB-induced resistance.

Our observation that 4HPR increased the levels of C16-dihydroceramide, the substrate of desaturase in the *de novo* SL biosynthesis pathway (Fig. 1), is consistent with the report showing 4HPR-induced inhibition of the enzyme (15). Our present findings that PDT-induced increased ceramides and C16-dihydroceramide in cells undergoing apoptosis are similar to our previous results in SCCVII mouse squamous carcinoma cells (18,32). Here we also demonstrate that combining PDT with 4HPR enhances C16-dihydroceramide levels, apoptosis and loss of clonogenicity. In addition, total ceramide levels, as well as most of the individual ceramides remained above baseline levels after PDT+4HPR. These data support the notion that dihydroceramide, together with ceramide, has a pro-apoptotic/pro-death role.

We demonstrate in the present report that PDT+4HPR-induced enhanced killing of SCC17B cells is associated with ER-localized and FB-sensitive *de novo* SL biosynthesis. This is in accordance with previous findings for PDT and 4HPR itself (15,16,29). Although each treatment alone increased mitochondrial dihydroceramide/ceramide accumulation in a FB-sensitive manner, combining PDT with 4HPR did not affect the process. The ER and mitochondria are connected by the mitochondria-associated membrane. Ceramide synthase activation has been shown in mitochondria-associated membrane after radiation (33). Reportedly, 4HPR can activate ceramide synthase (29). It remains to be established whether and at what subcellular site ceramide synthase is activated after PDT+4HPR.

We believe that our findings have important clinical implications. Combining PDT with another anticancer agent is advantageous because lower doses of individual treatments (LD20; Table I) are required for improved therapeutic benefit. This study also shows that combining PDT with 4HPR enhances cell killing via the *de novo* SL biosynthesis and mitochondrial apoptotic pathway. Thus, targeting these pathways could augment the therapeutic value of PDT.

## Figures and Tables

**Table I:** PDT+4HPR-induced augmented cell killing is FB-, zVAD- and ABT-sensitive in SCC17B cells.

Treatment	% Survival
FB	96±0.9
zVAD	96±0.8
ABT	97±1.3
4HPR	80±0.6^a^
4HPR+FB	91±0.9^a^,^b^
4HPR+zVAD	95±1.0^b^
4HPR+ABT	73±0.7^a^,^b^
PDT	79±0.6^a^
PDT+FB	93±0.7^b^
PDT+zVAD	91±1.3^a^,^b^
PDT+ABT	67±2.2^a^,^b^
PDT+4HPR	37±0.7^a^,^c^
PDT+4HPR+FB	73±1.7^a^,^b^
PDT+4HPR+zVAD	91±1.6^b^
PDT+4HPR+ABT	32±0.9^a^,^b^

FB, zVAD (10 μM each) and ABT (0.1 μM) were added 1 h prior to PDT (20 nM Pc4+200 mJ/cm^2^), 4HPR (2.5 μM) or the combination. Colonies were stained with crystal violet (0.1%) and counted 14 days after treatments. The data are shown as the average ± SEM (n=3–18 samples). Significant differences are shown between:

a treatment and untreated control;

b (treatment + inhibitor) and treatment;

c combination and individual treatments.

**Table II:** Effect of PDT±4HPR on individual ceramides in SCC17B cells.

Ceramide	Con	4HPR	PDT	PDT+4HPR
C14-ceramide	16.1±0.9	16.7±1.8	29.4±1.7^a^	22.6±0.8^a^,^d^
C16-ceramide	91.0±11.9	51.7±6.4^a^	129.6±8.5^a^	93.5±3.6^d^
C18-ceramide	17.8±2.1	21.5±3.0	61.6±4.2^a^	48.3±0.8^a^,^d^
C18:1-ceramide	7.6±0.3	10.8±1.1	25.5±2.6^a^	22.4±0.8^a^,^b^
C20-ceramide	5.2±0.7	9.1±0.5	21.4±1.8^a^	17.6±1.2^a^,^b^
C20:1-ceramide	1.5±0.2	2.3±0.2	5.3±0.6^a^	3.9±0.3^a^
C22-ceramide	51.6±2.7	37.5±3.2	132.7±10.6^a^	100.3±3.0^a^,^d^
C22:1-ceramide	18.7±1.7	16.1±1.9	49.6±4.5^a^	34.6±1.5^a^,^d^
C24-ceramide	161.9±9.1	97.7±5.1	252.6±22.4^a^	175.2±8.9^d^
C24:1-ceramide	199.3±9.4	110.0±8.6^a^	305.4±28.6^a^	196.7±9.0^d^
C26-ceramide	15.8±3.6	10.6±0.8	13.9±1.3	11.0±1.0
C26:1-ceramide	33.4±3.2	20.1±1.8^a^	33.5±2.3	23.0±2.3^a^,^c^

SCC17B cells were treated with PDT (20 nM Pc4+200 mJ/cm^2^), 4HPR (2.5 μM) or the combination, incubated for 10 h, collected, and processed for MS. Ceramide levels were calculated as pmoles/mg protein and are shown as the average ± SEM (n=3–4). Significant differences are shown between:

a treatment and untreated control;

b combination and 4HPR;

c combination and PDT;

d combination and both individual treatments. Con, untreated control. PDT alone data from ref. 16.
